# Retention Modelling of Phenoxy Acid Herbicides in Reversed-Phase HPLC under Gradient Elution

**DOI:** 10.3390/molecules25061262

**Published:** 2020-03-11

**Authors:** Alessandra Biancolillo, Maria Anna Maggi, Sebastian Bassi, Federico Marini, Angelo Antonio D’Archivio

**Affiliations:** 1 Dipartimento di Scienze Fisiche e Chimiche, Università degli Studi dell’Aquila, Via Vetoio, Coppito, 67100 L’Aquila (AQ), Italy; alessandra.biancolillo@univaq.it; 2 Hortus Novus srl, Via Campo Sportivo 2, Canistro, 67100 L’Aquila, Italy; maria.maggi79@gmail.com; 3 Dipartimento di Chimica, Università degli Studi di Roma “La Sapienza”, Piazzale Aldo Moro 5, 00185 Roma, Italy; sebastian.bassi@hotmail.it (S.B.); federico.marini@uniroma1.it (F.M.)

**Keywords:** phenoxy acid herbicides, HPLC, gradient elution, molecular descriptors, retention prediction, PLS regression

## Abstract

Phenoxy acid herbicides are used worldwide and are potential contaminants of drinking water. Reversed phase high-performance liquid chromatography (RP-HPLC) is commonly used to monitor phenoxy acid herbicides in water samples. RP-HPLC retention of phenoxy acids is affected by both mobile phase composition and pH, but the synergic effect of these two factors, which is also dependent on the structure and pKa of solutes, cannot be easily predicted. In this paper, to support the setup of RP-HPLC analysis of phenoxy acids under application of linear mobile phase gradients we modelled the simultaneous effect of the molecular structure and the elution conditions (pH, initial acetonitrile content in the eluent and gradient slope) on the retention of the solutes. In particular, the chromatographic conditions and the molecular descriptors collected on the analyzed compounds were used to estimate the retention factor k by Partial Least Squares (PLS) regression. Eventually, a variable selection approach, Genetic Algorithms, was used to reduce the model complexity and allow an easier interpretation. The PLS model calibrated on the retention data of 15 solutes and successively tested on three external analytes provided satisfying and reliable results.

## 1. Introduction

Phenoxy acid herbicides are synthetic analogues of the plant regulator auxins and are extensively used to control broad-leaved weeds in many crops, such as rice, wheat and soybeans [1]. The treatment by phenoxy acids induces “auxin overdose” into the unwanted vegetal species that undergoing disorganized and uncontrolled growth eventually die. 2,4-dichlorophenoxyacetic acid (2,4-D), although first marketed in 1944, is still one of the most widely used herbicides in the world, together with the parent products 2-(4-chloro-2-methylphenoxy) propanoic acid (mecoprop), (4-chloro-2-methylphenoxy)acetic acid (MCPA) and 2-(2,4-dichlorophenoxy)propanoic acid (dichloroprop) [2,3]. Phenoxy acids are highly soluble in the aqueous media and therefore, can easily migrate through the agricultural environment finally leading to groundwater contamination [4,5], which can compromise drinking water quality and affect human health because of chronic toxicity, endocrine-disrupting action and potential carcinogenic effects [6,7,8]. Phenoxy acids in water or food can be detected and quantified by reversed-phase high-performance liquid chromatography (RP-HPLC) coupled to spectrophotometric or mass-spectrometry detection [3,9,10,11]. Multi-residue determination in real samples by RP-HPLC is usually preceded by a procedure for concentration and isolation of the analytes, such as liquid-phase micro-extraction or solid-phase extraction [12], but differently from gas-chromatography [13,14], preliminary derivatization of the phenoxy acids is not needed.

Routine monitoring of trace pollutants in water requests chromatographic methods able to simultaneously detect and quantify many structural congeners or multi-class mixtures. In this context, the empirical trial-and-error strategies still used to develop chromatographic methods may be inefficient or excessively time-consuming. To overcome such limitation various multivariate statistical approaches have been proposed [15,16,17,18] with the aim of providing predictive tools for the chromatographic retention. A widely used approach is quantitative structure-retention relationship (QSRR) methodology [19,20] which is aimed at establishing a relationship between the solute structure, encoded by a set of molecular descriptors, and the retention time or a related parameter. Approaches complementary to QSRR, on the other hand, are applied to model the effects of the separation conditions on the chromatographic behavior of a target solute or a mixture of analytes. To this end, multivariate design of experiments combined with response surface methodology can be employed to optimize the chromatographic response(s) of interest [21,22,23,24,25], such as retention time, pair or global resolution and overall analysis time. Apart from the above two kinds of predictive approaches, which can be considered orthogonal according to the complementary nature of the described variability, QSRR-based models combining both molecular descriptors of solutes and the features of the eluent, the column or both have been proposed [26,27,28,29,30]. These comprehensive models, once calibrated on a pool of known solutes and a well-designed set of separation conditions, allow to predict the behavior of unseen molecules in the whole experimental domain covered in calibration or to transfer retention data among different columns [31,32,33,34]. 

In RP-HPLC the composition of the eluent, mostly a binary aqueous mixture, is the experimental parameter commonly tuned to find the optimal separation conditions. Moreover, to overcome the typical disadvantages of isocratic elution, such as poor resolution of early peaks, broadening of late peaks, band tailing, and long separation times, mobile-phase gradients are required [35,36], especially when dealing with complex mixtures or structural congeners. The concentration of organic solvent in the aqueous mobile phase is usually increased during the chromatographic run, which determines a progressive increase of the elution power of the eluent and a consequent decrease in solute retention. Apart from composition, the retention behavior of weak acids is also influenced by the eluent pH, that must be fixed or properly modulated during the chromatographic run [37]. An increase in pH of the mobile phase produces a progressive ionization of the solute and a consequent decrease in its retention time. It must be remarked that the organic modifier of the mobile phase affects the acidity of both medium and solutes, which does not allow to easily deduce the synergic effect of the eluent pH and composition on the retention behavior of the target analytes, especially when gradient elution is applied.

In a previous work [38], we modelled the combined effect of pH and composition of water-acetonitrile eluent on the retention of phenoxy acids and structurally related carboxylic acids under isocratic elution conditions. The molecular structure of the phenoxy acids was described by a-priori selected quantum-chemical properties (dipole moment, mean polarizability, anisotropy of polarizability, water/n-heptane partition coefficient and a hydrogen bond descriptor) of both neutral and ionized forms together with the experimental pKa, while pH and acetonitrile volume fraction were the variables associated with the eluent. A three-layer artificial neural network was used to generate the retention model. In the present work, we developed a comprehensive model to predict the retention behavior of phenoxy acid herbicides and related carboxylic acids under the application of linear composition gradients at different pH levels. Moreover, the molecular structure of solutes is encoded by non-quantum-chemical theoretical descriptors provided by popular software *Dragon* (Dragon 6, Talete srl, Milan) [39] that, as compared to quantum-chemical molecular properties, are quickly accessible to users with no specific expertise in theoretical chemistry. The regression problem was solved by Partial Least Square [40,41], as it is one of the most commonly applied regression methods in this context [20,42,43]. Eventually, Genetic Algorithms (GA) [44] were applied in order to reduce the model complexity and make its interpretation easier. 

## 2. Results

The set of solutes investigated in this work (reported in Table A1) consists of eight phenoxy acid herbicides (2,4-D, 2,4,5-T, 2,4,5-TP, MCPA, dichlorprop, mecoprop, clopyralid and trichlopyr) and ten derivatives of benzoic acid, phenylacetic acid and phenoxyacetic acid, pKa of these compounds ranging between 2.29 and 4.31. The RP-HPLC retention data of the 18 analytes were collected at three different values of eluent pH, namely 2, 3 and 4. For each pH level the starting acetonitrile volume fraction φ_i_ was set to 30, 40, 50, 60 and 70%. While pH of the mobile phase was kept fixed during elution, the acetonitrile content was fixed at the starting value (isocratic elution) or linearly increased to 100% in 15, 20 or 25 min. The descriptors selected to identify the various elution modes were the eluent pH, φ_i_ and the gradient slope ϕ defined as (100−φ_i_)/t_g_, where t_g_ was the application time of the linear gradient, and ϕ = 0 for the isocratic condition. The chromatographic conditions are resumed in Table 1. Nevertheless, in the chromatograms collected under application of the 60 elution modes obtained by the combination of the above designed levels for the three eluent descriptors, we did not consider those peaks given by both poorly or strongly retained compounds. 753 retention time (t_r_) values were finally collected and the (base 10) logarithm of the related retention factor k [45,46,47], defined as k = (t_r_−t_0_)/t_0_, t_0_ being the column’s dead time, was assumed as the model response. The use of the logarithm of the retention factor has been preferred to the retention factor itself because it can be directly related to some physico-chemical parameters governing the retention process. In particular, Ciura et al. [45] claim that the use of log k as dependent variable in QSRR presents various advantages, one of the most relevant of which being that it can be used as a hydrophobicity index. Similar considerations are reported by Kaliszan and collaborators [46] who showed how log k could be linearly related to the fraction of the organic modifier in the mobile phase. 

### 2.1. Prediction of the Retention Factor k

**Figure 1:**
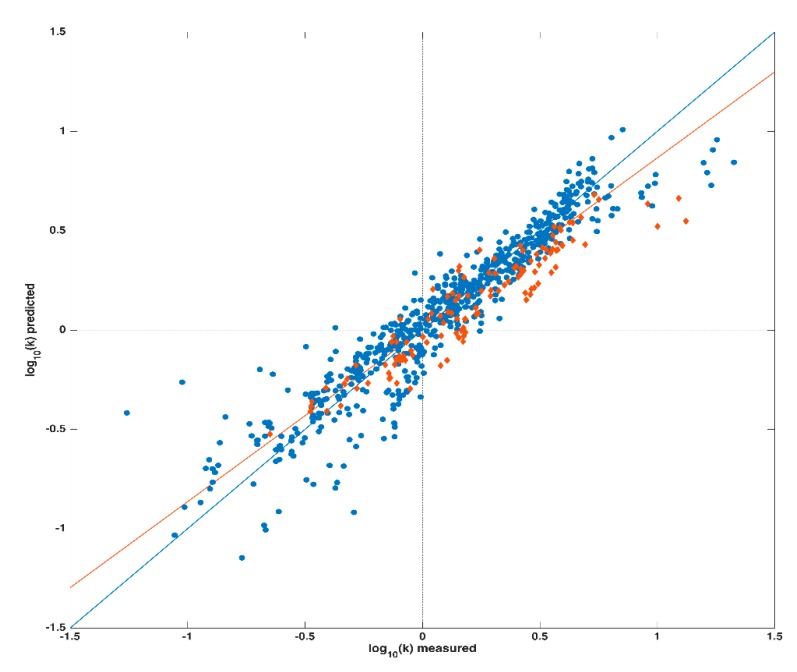
PLS model. Legend: Calibration set: blue dots; Test set: orange diamonds.

Prior to the creation of any prediction model, the three chromatographic parameters (pH, φ_i_ and ϕ) and the 329 descriptors were organized in a unique data matrix X. Then, in order to externally validate the regression model, data collected on the 18 compounds were divided into a training set (for building the calibration model), and a test set (for validation); in particular, the latter one was constituted by data collected on 2,4-diclorofenossiacetic acid, trichlopyr and 2-iodobenzoic acid. As mentioned above, a PLS regression model was created (exploiting only the training set) in order to fit the logarithm of the retention factor k to the autoscaled data matrix X. The calibration model, calculated extracting 11 latent variables (LVs) (defined into a cross-validation procedure), provided a coefficient of determination (R_cv_^2^) of 0.887 and a Root Mean Square Error in Cross-Validation (RMSECV) of 0.142. The application of this model to the test set provided a R_test_^2^ of 0.872 and a Root Mean Square Error in Prediction (RMSEP) of 0.156; a graphical representation of these results is displayed in Figure 1 showing the agreement between computed/predicted and experimental responses. The black solid line in the plot depicts the ideal fitting (i.e., RMSEP = 0), while the red one represents the actual one; as it can be seen from the good overlap between the two straight lines, and in accordance with the R_cv_^2^ and R_test_^2^ values shown, the model properly fits the data under study. 

Eventually, despite the results provided by the PLS model were quite satisfying, a variable selection approach, Leardi’s Genetic Algorithm (GA) [44], was tested aiming at two final goals: evaluating whether variable selection could improve the predictive accuracy and simplifying the interpretation of the regression model. 

The genetic algorithm was applied to the training set (with the parameters reported in Table A2 in Appendix A) and the R_cv_^2^ (5 cancelation groups) was used as fitness.

The predictors were sorted according to the frequency of selection in the 100 GA runs and PLS models with an increasing number of variables were built according to a forward stepwise scheme: the model leading to the highest R_cv_^2^ (5 cancelation groups), which included 29 predictors (see Table 2), was selected as the final one. This model was built on 4 latent variables and resulted in a RMSEC = 0.143 and a RMSECV = 0.148 (corresponding to R^2^ = 0.883 and R_cv_^2^ = 0.876, respectively). The model was then applied to the test molecules for the final (external) validation and resulted in a RMSEP of 0.167, corresponding to a R^2^_pred_ of 0.847. This means that, in the present case, the use of a variable selection approach mainly helps the interpretation of the regression model in chemical terms, rather than leading to an improvement in the prediction accuracy. Nevertheless, it has to be emphasized that the results obtained are comparable with those of similar works on QSRR published in the literature (for instance [48] where models (built on log k) have in general SEP values between 0.16 and 0.22, and R^2^_pred_ around 0.75–0.88), and, framed into a wider scenario, are in line with studies on experimentally measuring retention factor, considering the uncertainty associated to the estimation of this entity [49]. 

## 3. Discussion

### Interpretation of the Model

The PLS model discussed in Section 2.1 provided satisfying and reliable results, indicating that pH, φ_i_, ϕ, and the inspected molecular descriptors represent a reasonable set of predictors for the estimation of log(k) for the investigated phenoxy acid herbicides and related carboxylic acids. The regression coefficients from this model are reported in Table A3. 

The application of GA leads to a deeper insight into the system under study; in fact, it provides information about the contributions of the 29 selected variables. 

**Figure 2:**
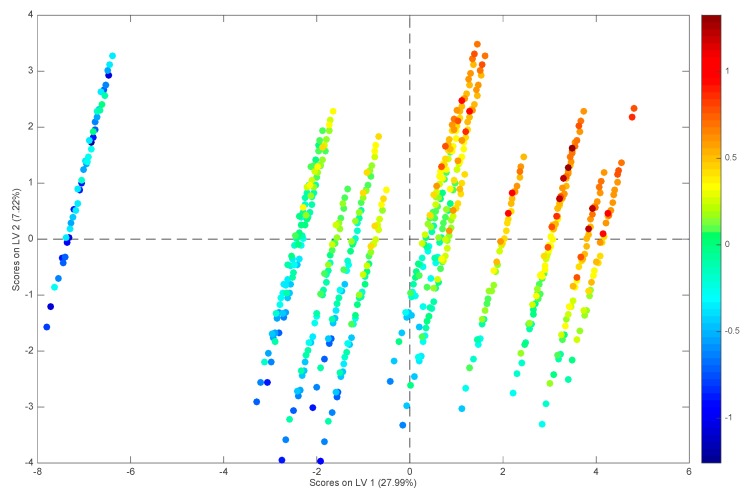
PLS model built on the reduced set of 29 predictors selected by GA: scores plot of the training samples. The points are colored according to their values of log k.

The sample scores along the first two latent variables of the model are reported in Figure 2, where their relation to the values of the response is also highlighted. By inspecting the scores in Figure 2, it is apparent how LV1 accounts mostly for the differences among substances while the mobile phase conditions are mainly responsible for the variance along LV2. In general, log k increases both along LV1 and along LV2. 

**Figure 3:**
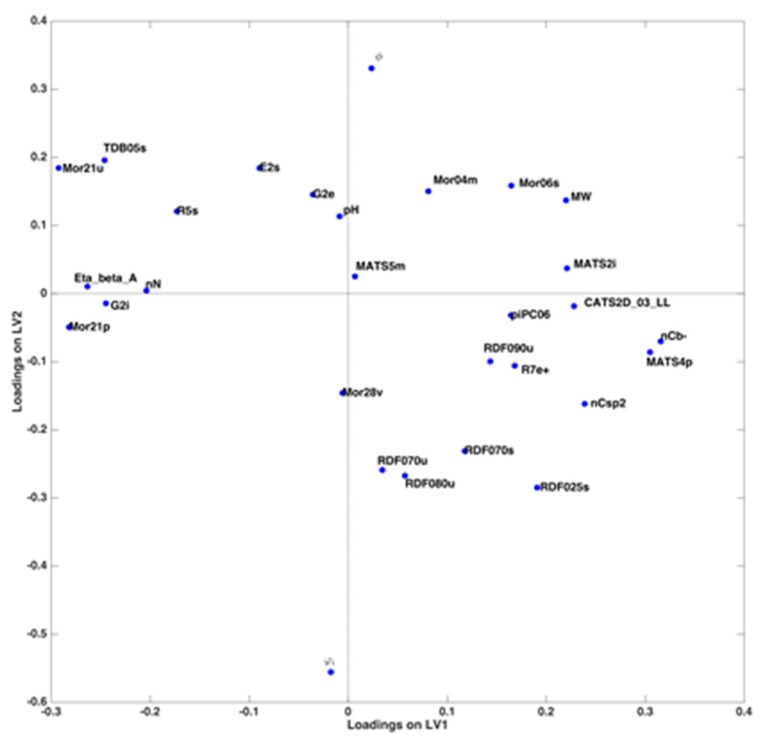
Variables loadings projected on the first two latent variables of the PLS model.

Investigation of the corresponding loadings (Figure 3) confirms what already evidenced by the scores plot and provides details for a comprehensive interpretation. Indeed, the three predictors related to the mobile phase conditions have practically zero loadings along LV1, thus confirming that the latent variable is affected only by the molecular descriptors characterizing the analytes. In particular, MATS2i, nCb-, MATS4p, MW, nCsp2 and CATS2D_03_LL have the highest positive loadings, while Mor21u, Eta_beta_A, TDB05s, G2i, nN and Mor21p are those giving the largest negative contribution. This means that the value of log k is higher for molecules having higher values of the former descriptors and lower values of the latter, and vice versa.

On the other hand, LV2 is characterized by a very negative contribution of the % of acetonitrile and a highly positive loading of the slope of the gradient; pH of the mobile phase also has a positive, although lower, loading on this component. For each substance, the value of log k increases when a lower initial amount of acetonitrile is used, together with a steeper slope of the gradient; a more basic pH also contributes to increase the value of the retention factor. 

In summary, the PLS regression model discussed in Section 2.1 provided satisfying and reliable results, indicating that pH, φ_i_ ϕ, and the inspected molecular descriptors represent a reasonable set of predictors for the estimation of log k for the investigated herbicides. The use of a variable selection approach in this specific case did not lead to an improvement in the prediction accuracy but was useful for the interpretation of the regression model in chemical terms.

## 4. Materials and Methods 

### 4.1. Chemicals

Pestanal standards (purity greater than 99%) of the phenoxy acid herbicides 2-(4-chloro-2-methylphenoxy) propanoic acid (mecoprop), 2,4-dichlorophenoxyacetic acid (2,4-D), (4-chloro-2-methylphenoxy)acetic acid (MCPA), (2,4,5-trichlorophenoxy) acetic acid (2,4,5-T), 2-(2,4,5-trichlorophenoxy)propanoic acid (2,4,5-TP), 2,6-dichloro-pyridine-2-carboxylic acid (clopyralid), 2-(2,4-dichlorophenoxy)propanoic acid (dichloroprop) and [(3,5,6-trichloro-2-pyridinyl)oxy]acetic acid (triclopyr) were obtained from Labor Dr. Ehrenstorfer-Schäfers (Augsburg, Germany). Chemical standards of phenoxyacetic acid, benzoic acid and phenylacetic acid derivatives with purity greater than 95% were purchased from Sigma–Aldrich (St. Louis, MO, USA). Stock solutions (1.00 g L^−1^) of individual analytes were prepared by dissolving accurately weighed 10 mg of each solute in 10 mL of HPLC-grade acetonitrile (Carlo Erba Reagenti, Milano, Italy) and stored at 4 °C. HPLC-grade acetonitrile was acquired from Sigma-Aldrich (St. Louis, MO, USA). Double deionized water was obtained from a Milli-Q filtration/purification system (Millipore, Bedford, MA, USA).

### 4.2. HPLC Apparatus

Retention data were collected with a Waters chromatographic system (Waters, Milford, MA, USA) consisting of a Model 600 pump, a 600 Pump Controller Module, an Acqua 717 plus auto-sampler and a 996-photodiode array detector. Chromatographic data management was automated using the Empower data acquisition system (Waters). Eluent degassing was performed by an Agilent 1200 system (Agilent Technologies, Waldbronn, Germany). The analyses were carried on a 250 × 4.6 mm Kinetex C18 (Phenomenex, Torrance, CA, USA) analytical column preceded by a 4 × 3mm UHPLC guard cartridge (Phenomenex) both packed with octadecyl-silica having 5 μm particle size.

### 4.3. Collection of Retention Data

The HPLC analyses were carried out at room temperature using water-acetonitrile mobile phases with a flow-rate of 1mLmin^−1^. The retention time of the analytes was collected at fixed values of the eluent pH obtained by mixing the aqueous phase buffered at pH 2, 3 or 4. To control the acidity of the mobile phase a phosphate buffer at 0.1 M concentration was used. pH of water, before mixing with acetonitrile, was adjusted to the desired value by addition of HCl. pH measurements were carried out by means of an Orion 420 A (Beverly, MA, USA) pH meter equipped with an Orion 9107 electrode. Five levels for the initial acetonitrile volume fraction φ_i_ were considered, namely 30, 40, 50, 60 and 70%. For each combination of the eluent pH and φ_i_ pair, the acetonitrile content in the mobile phase was kept constant for the whole chromatographic run (isocratic elution) or was linearly increased until 100% in 15, 20, or 25 min. After gradient elution the column was re-equilibrated by fluxing the mobile phase at the initial composition for ten minutes before successive analysis. Retention data were collected by injecting 10 μL of acetonitrile solutions of single compounds (5mgL^−1^) or mixtures providing well-resolved chromatograms. The detection wavelength (220 nm for most analytes) was the one providing the maximum peak height. The dead time of the column was determined by injection of an aqueous solution of sodium nitrate.

### 4.4. Molecular Descriptors

Starting geometries of phenoxy acid herbicides and the other carboxylic acids were drawn by means of the MacroModel 7.1 molecular modelling program package [50]. The global energy minimum of each molecule was searched using the MM2 force field. Software Dragon 6 [39] was used to compute the molecular descriptors from the optimised geometries. The version utilised in this work provides 4885 descriptors classified as zero- (0D), one- (1D), two- (2D) and three-dimensional (3D) descriptors depending on the fact they are computed from the chemical formula, the substructure list representation, the molecular graph or the geometrical representation of the molecule, respectively. After removal of constant and highly correlated variables (r > 0.85), 329 molecular descriptors were retained for further analyses.

### 4.5. Development and Validation of Retention Models

The data matrix constituted of the three chromatographic parameters and the 329 molecular descriptors were used as predictors to estimate log k by Partial Least Squares (PLS) [40,41]. The regression model was built on a restricted sub-set of data (the training set) in order to pursue validation on the test set. The optimal complexity of the calibration model (i.e., the number of LVs to be extracted) were defined into a cross-validation procedure (not involving the test set). Once the PLS model parameters were defined (by inspection of the lowest RMSECV) it was validated by predicting the external test set. The goodness of the model was estimated by inspecting R_cv_^2^ and R_test_^2^.

In order to test whether feature reduction could improve the model from the prediction and the interpretation standpoint, Genetic Algorithms (GA) [44] were applied for variable selection. Concisely, this iterative approach starts picking random sets of variables, which, by means of genetic operators, lead to the generation of *offsprings*. Calculations are then re-run on the subsets of features, and the variables providing the most performant solution are retained. The procedure is repeated until the optimal solution is reached. For more details, the reader is addressed to [51].

All the calculations were run in Matlab 2015b (The Mathworks Inc., Natick, MA) using in-house routines or by the PLS toolbox Version 8.1 (Eigenvector Research, Inc.Manson, WA).

## Figures and Tables

**Table 1:** Chromatographic conditions utilized to collect the retention data.

Column	Kinetex C18 (Phenomenex)
Eluent	water-acetonitrile, flux:1 mLmin^−1^
Elution mode	Starting acetonitrile volume fraction (φ_i_): 30, 40, 50, 60, 70%
	Eluent pH: 2,3,4
	Application time of linear composition gradient (from φ_i_ to 100%): none, 15, 20, 25 min

**Table 2:** List of the 29 predictors selected by the GA in order of decreasing frequency.

Variable	Description	Block
φ_i_	starting acetonitrile volume fraction in the eluent	-
ϕ	gradient slope	-
pH	eluent pH	-
MATS2i	Moran autocorrelation of lag 2 weighted by ionization potential	2D autocorrelations
nCb-	number of substituted benzene C(sp2)	Functional group counts
Mor21u	signal 21/unweighted	3D-MoRSE descriptors
MATS4p	Moran autocorrelation of lag 4 weighted by polarizability	2D autocorrelations
Eta_beta_A	eta average VEM count	ETA indices
TDB05s	3D Topological distance-based descriptors - lag 5 weighted by I-state	3D autocorrelations
RDF070u	Radial Distribution Function - 070/unweighted	RDF descriptors
RDF025s	Radial Distribution Function - 025/weighted by I-state	RDF descriptors
Mor06s	signal 06/weighted by I-state	3D-MoRSE descriptors
G2i	2nd component symmetry directional WHIM index/weighted by ionization potential	WHIM descriptors
MW	molecular weight	Constitutional indices
nCsp2	number of sp2 hybridized Carbon atoms	Constitutional indices
MATS5m	Moran autocorrelation of lag 5 weighted by mass	2D autocorrelations
Mor28v	signal 28/weighted by van der Waals volume	3D-MoRSE descriptors
R7e+	R maximal autocorrelation of lag 7/weighted by Sanderson electronegativity	GETAWAY descriptors
nN	number of Nitrogen atoms	Constitutional indices
piPC06	molecular multiple path count of order 6	Walk and path counts
RDF080u	Radial Distribution Function - 080/unweighted	RDF descriptors
RDF090u	Radial Distribution Function - 090/unweighted	RDF descriptors
RDF070s	Radial Distribution Function - 070/weighted by I-state	RDF descriptors
Mor04m	signal 04/weighted by mass	3D-MoRSE descriptors
Mor21p	signal 21/weighted by polarizability	3D-MoRSE descriptors
G2e	2nd component symmetry directional WHIM index/weighted by Sanderson electronegativity	WHIM descriptors
E2s	2nd component accessibility directional WHIM index/weighted by I-state	WHIM descriptors
R5s	R autocorrelation of lag 5/weighted by I-state	GETAWAY descriptors
CATS2D_03_LL	Lipophilic-Lipophilic at lag 03	CATS2D

## Appendix A

**Table A1:** Molecular structure and pKa value (from literature [38]) of the investigated acids.

Name	Structure	pKa
2,4-D		2.73
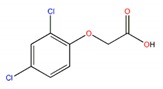
2,4,5-T		2.83
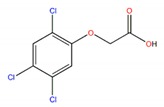
2,4,5-TP		2.84
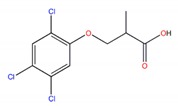
Clopyralid		2.29
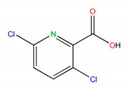
Dichlorprop		3.10
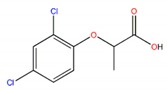
MCPA		3.13
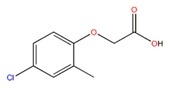
Mecoprop		3.10
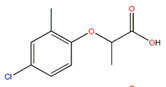
Tryclopir		3.97
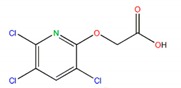
Benzoic acid		4.19
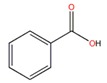
Salicylic acid		2.97
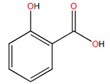
2-Iodobenzoic acid		2.93
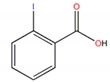
3-Iodobenzoic acid		3.85
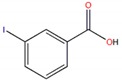
4-Iodobenzoic acid		4.00
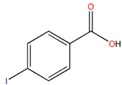
Phenylacetic acid		4.31
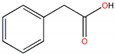
4-Chlorophenylacetic acid		2.76
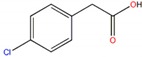
4-Nitrophenylacetic acid		3.85
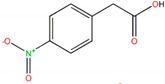
Phenoxyacetic acid		3.17
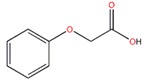
4-Chlorophenoxyacetic acid		3.56
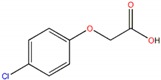

**Table A2:** Parameters of the genetic algorithm.

Parameters	Value
Number of chromosomes	30
Probability of selection in the original population	0.015
Maximum number of variables per chromosome	30
Probability of mutation	0.01
Probability of cross-over	0.50
Backward stepwise selection every	100 iterations
Number of runs	100
Number of evaluations per run	200

**Table A3:** Regression coefficients extracted from the PLS model based on the 29 descriptors selected by GA.

Descriptor	Coefficient
φ_i_	−0.686
ϕ	−0.091
pH	0.212
MATS2i	0.061
nCb-	0.097
Mor21u	−0.067
MATS4p	0.080
Eta_beta_A	−0.073
TDB05s	−0.042
RDF070u	−0.025
RDF025s	0.0366
Mor06s	0.0900
G2i	−0.0400
MW	0.093
nCsp2	0.039
MATS5m	0.008
Mor28v	0.001
R7e+	0.034
nN	−0.048
piPC06	0.0658
RDF080u	−0.015
RDF090u	0.0188
RDF070s	0.016
Mor04m	0,025
Mor21p	−0.081
G2e	−0.007
E2s	0.025
R5s	−0.037
CATS2D_03_LL	0.0300
